# Drugs to Treat Systemic Lupus Erythematosus: Relationship between Current Use and Cardiovascular Risk Factors

**DOI:** 10.1111/j.1753-5174.2007.00004.x

**Published:** 2008-07

**Authors:** Young Hee Rho, Annette Oeser, Cecilia P Chung, Jason D Morrow, C Michael Stein

**Affiliations:** * Department of Medicine, Vanderbilt University School of MedicineNashville, TN, USA; † Department of Pharmacology, Vanderbilt University School of MedicineNashville, TN, USA

**Keywords:** Prednisone, Hydroxychloroquine, NSAID, COX-2 Inhibitor, Azathioprine, Methotrexate

## Abstract

**Objectives:**

Cardiovascular risk is increased in patients with systemic lupus erythematosus (SLE). Drugs used to treat SLE can modify traditional cardiovascular risk factors. We examined the effect of selected drugs used in the treatment of SLE on cardiovascular risk factors.

**Methods:**

We compared systolic and diastolic blood pressure, serum lipid concentrations, glucose, homocysteine, and urinary F_2_-isoprostane concentrations in 99 patients with lupus who were either current users or non-users of systemic corticosteroids, antimalarials, non-steroidal anti-inflammatory drugs (NSAIDs), COX-2 selective NSAIDs, azathioprine, and methotrexate. Multivariable adjustment was done with linear regression modeling using sex, age and disease activity (SLEDAI) as controlling variables.

**Results:**

Serum triglyceride concentrations were higher (135.1 ± 61.4 vs. 95.3 ± 47.5 mg/dL, adjusted *P* = 0.003) in patients receiving corticosteroids. Homocysteine concentrations were marginally higher in patients receiving methotrexate (adjusted *P* = 0.08). Current use of either NSAIDs or COX-2 inhibitors was not associated with increased cardiovascular risk factors. Current hydroxychloroquine use was not associated with significant alterations in lipid profiles.

**Conclusions:**

In a non-random sample of patients with SLE, current corticosteroid use was associated with increased triglyceride concentrations, but other drugs had little effect on traditional cardiovascular risk factors.

## Introduction

Patients with systemic lupus erythematosus (SLE) have accelerated atherosclerosis and increased cardiovascular morbidity and mortality [1]. We have found that cardiovascular risk factors such as triglyceride concentrations and the prevalence of hypertension were significantly higher in patients with SLE than controls [2]. Disease characteristics, or drugs used to treat the disease, could contribute to alterations in cardiovascular risk factors. Drugs used to treat SLE such as corticosteroids, non-steroidal anti-inflammatory drugs (NSAIDs) and COX-2 inhibitors can adversely affect cardiovascular risk factors such as blood pressure and lipid concentrations [3–5]. On the other hand, hydroxychloroquine has been reported to decrease total and LDL cholesterol and triglyceride concentrations [6]. We examined the hypothesis that current use of drugs used to treat SLE affects cardiovascular risk factors.

## Methods

This study enrolled 99 patients with SLE from the Vanderbilt Lupus Cohort. This cohort had an increased prevalence of coronary atherosclerosis detected by electron-beam computed tomography (EBCT). The methods have been reported in detail elsewhere [2,7–9]. All subjects gave written informed consent and the study was approved by the Institutional Review Board of Vanderbilt University Hospital. All patients met the 1997 American College of Rheumatology (ACR) classification criteria for SLE [10]. Patients with pre-existing cardiovascular disease such as angina, stroke or myocardial infarction and those receiving lipid-lowering agents were excluded. Demographic and disease characteristics and cardiovascular risk factors including systolic and diastolic blood pressure, serum HDL and LDL cholesterol, triglycerides, glucose, serum homocysteine, and urine F_2_-isoprostane concentrations, a measure of oxidative stress [11], were measured.

## Statistical Analysis

Cardiovascular risk factors were compared in patients receiving or not receiving a drug of interest (systemic corticosteroids, antimalarials, NSAIDs, COX-2 inhibitors, azathioprine, and methotrexate) using Mann-Whitney U tests. Additional multivariable adjustment was performed by linear regression using age, sex and SLEDAI (Systemic Lupus Erythematosus Disease Activity Index) [12] as controlling variables. F_2_-isoprostane concentrations were logarithmic-transformed for regression analysis. These analyses were exploratory, therefore no adjustment for multiple statistical comparisons were performed. Statistical analysis was conducted using SPSS 15.0 for Windows (SPSS Inc., Chicago, USA). Descriptive values are presented as mean ± standard deviation (SD). Statistical significance was determined as *P* < 0.05.

## Results

Descriptive data for the SLE population are shown in Table 1. Cardiovascular risk factors in patients receiving or not receiving the drugs of interest are shown in Tables 2–7. As shown in Table 2, corticosteroid use was associated with increased serum triglycerides even after adjustment for age, sex, and disease activity (SLEDAI) (*P* = 0.003). Antimalarial use was not associated with altered cardiovascular risk factors. Neither NSAIDs nor COX-2 inhibitors were associated with increased blood pressure or other risk factors. When patients taking (31 patients, 9 ACEI users) or not taking antihypertensive drugs were analyzed separately, blood pressure did not differ between non-NSAID and NSAID users (non-antihypertensive drug users: systolic 116.4 ± 13.4 mm Hg vs. 115.7 ± 13.8 mm Hg, *P* = 0.85; diastolic 72.5 ± 10.7 mm Hg vs. 70.9 ± 12.5 mm Hg, *P* = 0.68; anti-hypertensive users: systolic 126.5 ± 24.8 mm Hg vs. 129.5 ± 14.2 mm Hg, *P* = 0.50; diastolic 78.6 ± 18.9 mm Hg vs. 71.8 ± 14.4 mm Hg, *P* = 0.45). COX-2 inhibitors were weakly associated with lower serum triglyceride concentrations and F_2_-isoprostane excretion after statistical adjustment (*P* = 0.02 and *P* = 0.03, Table 5). Methotrexate use was associated with higher serum homocysteine concentrations (unadjusted *P* = 0.03) but this was attenuated after statistical adjustment (*P* = 0.08, Table 7). Systolic blood pressure and triglyceride concentrations also tended to be higher in patients receiving methotrexate.

**Table 1:** Descriptive data for the study group (N = 99)

Factor	Mean ± SD
Age (years)	40.2 ± 11.4
Sex (M/F)*	8/91
SLEDAI	4.3 ± 4.0
SLICC	0.79 ± 1.2
Systolic BP (mm Hg)	119.6 ± 17.7
Diastolic BP (mm Hg)	73.8 ± 13.8
HDL cholesterol (mg/dL)	48.0 ± 14.8
LDL cholesterol (mg/dL)	105.4 ± 37.3
Triglycerides (mg/dL)	119.4 ± 59.4
Glucose (mg/dL)	87.1 ± 27.0
Homocysteine (umol/L)	9.29 ± 2.78
F_2_ Isoprostane (ng/mg Creatinine)	2.84 ± 2.36

* Shown as frequency.

SD = standard deviation; SLEDAI = systemic lupus erythematosus disease activity index; SLICC = systemic lupus international collaborating clinics group damage index; BP = blood pressure.

**Table 2:** Cardiovascular risk factors and current corticosteroid use

Factor	Corticosteroids (N = 60)	No corticosteroids (N = 39)	*P*	*P* (Adjusted)
Systolic BP (mm Hg)	121.7 ± 19.1	116.3 ± 15.0	0.14	0.40
Diastolic BP (mm Hg)	75.8 ± 14.4	70.8 ± 12.6	0.04	0.16
HDL cholesterol (mg/dL)	48.9 ± 16.0	46.5 ± 12.8	0.68	0.25
LDL cholesterol (mg/dL)	108.9 ± 43.1	100.1 ± 25.6	0.31	0.41
Triglycerides (mg/dL)	135.1 ± 61.4	95.3 ± 47.5	0.001	0.003
Glucose (mg/dL)	88.6 ± 33.1	84.9 ± 13.1	0.70	0.67
Homocysteine (umol/L)	9.78 ± 2.98	8.55 ± 2.31	0.05	0.11
F_2_-Isoprostane (ng/mg Creatinine)	2.82 ± 2.31	2.87 ± 2.47	0.88	0.96

*P* = unadjusted *P* values; *P* (adjusted) = adjusted for age, sex, and SLEDAI. Data are shown as mean ± SD.

**Table 3:** Cardiovascular risk factors and current antimalarial use

Factor	Antimalarials (N = 64)	No antimalarials (N = 35)	*P*	*P* (Adjusted)
Systolic BP (mm Hg)	118.7 ± 15.0	121.2 ± 22.0	0.97	0.59
Diastolic BP (mm Hg)	73.3 ± 12.3	74.7 ± 16.4	0.75	0.68
HDL cholesterol (mg/dL)	48.1 ± 13.2	47.7 ± 17.4	0.59	0.62
LDL cholesterol (mg/dL)	108.2 ± 39.4	100.3 ± 33.1	0.69	0.23
Triglycerides (mg/dL)	117.1 ± 63.2	123.6 ± 52.2	0.25	0.52
Glucose (mg/dL)	85.3 ± 19.3	90.4 ± 37.3	0.46	0.46
Homocysteine(umol/L)	9.27 ± 2.70	9.33 ± 2.97	0.89	0.96
F_2_ Isoprostane (ng/mg Creatinine)	2.66 ± 2.23	3.13 ± 2.56	0.48	0.46

*P* = unadjusted *P* values; *P* (adjusted) = adjusted for age, sex, and SLEDAI. Data are shown as mean ± SD.

**Table 4:** Cardiovascular risk factors and current NSAID use

Factor	NSAIDs (N = 22)	No NSAIDs (N = 77)	*P*	*P* (Adjusted)
Systolic BP (mm Hg)	118.8 ± 14.8	119.8 ± 18.5	0.87	0.85
Diastolic BP (mm Hg)	71.1 ± 12.6	74.6 ± 14.2	0.39	0.42
HDL cholesterol (mg/dL)	50.0 ± 16.6	47.4 ± 14.3	0.46	0.78
LDL cholesterol (mg/dL)	107.4 ± 31.6	104.8 ± 39.0	0.37	0.72
Triglycerides (mg/dL)	101.8 ± 47.6	124.4 ± 61.7	0.12	0.11
Glucose (mg/dL)	86.6 ± 16.8	87.3 ± 29.3	0.68	0.71
Homocysteine(umol/L)	9.19 ± 2.32	9.32 ± 2.92	0.81	0.88
F_2_ Isoprostane (ng/mg Creatinine)	3.10 ± 1.94	2.75 ± 2.49	0.13	0.23

*P* = unadjusted *P* values; *P* (adjusted) = adjusted for age, sex, and SLEDAI. Data are shown as mean ± SD.

**Table 5:** Cardiovascular risk factors and current COX-2 inhibitor use

Factor	COX-2 (N = 11)	No COX-2 (N = 88)	*P*	*P* (Adjusted)
Systolic BP (mm Hg)	118.7 ± 15.3	120.0 ± 18.1	0.97	0.72
Diastolic BP (mm Hg)	73.1 ± 9.3	73.9 ± 14.3	0.98	0.85
HDL cholesterol (mg/dL)	51.9 ± 12.1	47.5 ± 15.0	0.23	0.39
LDL cholesterol (mg/dL)	91.5 ± 25.6	107.1 ± 38.3	0.14	0.17
Triglycerides (mg/dL)	83.5 ± 50.2	123.9 ± 59.1	0.01	0.021
Glucose (mg/dL)	91.6 ± 21.7	86.6 ± 27.6	0.25	0.70
Homocysteine(umol/L)	8.58 ± 2.37	9.38 ± 2.83	0.31	0.29
F_2_ Isoprostane (ng/mg Creatinine)	1.36 ± 0.71	2.98 ± 2.41	0.06	0.03

*P* = unadjusted *P* values; *P* (adjusted) = adjusted for age, sex, and SLEDAI. Data are shown as mean ± SD.

**Table 6:** Cardiovascular risk factors and current azathioprine use

Factor	Azathioprine (N = 10)	No Azathioprine (N = 89)	*P*	*P* (Adjusted)
Systolic BP (mm Hg)	117.9 ± 18.3	119.8 ± 17.7	0.83	0.53
Diastolic BP (mm Hg)	66.7 ± 16.1	74.6 ± 13.4	0.16	0.034
HDL cholesterol (mg/dL)	53.9 ± 15.0	47.3 ± 14.7	0.15	0.09
LDL cholesterol (mg/dL)	89.3 ± 26.2	107.2 ± 38.0	0.09	0.09
Triglycerides (mg/dL)	98.9 ± 39.7	121.7 ± 60.9	0.37	0.22
Glucose (mg/dL)	84.3 ± 11.4	87.5 ± 28.2	0.81	0.77
Homocysteine(umol/L)	9.62 ± 3.40	9.25 ± 2.72	0.90	0.90
F_2_ Isoprostane (ng/mg Creatinine)	3.38 ± 1.78	2.78 ± 2.42	0.21	0.16

*P* = unadjusted *P* values; *P* (adjusted) = adjusted for age, sex, and SLEDAI. Data are shown as mean ± SD.

**Table 7:** Cardiovascular risk factors and current methotrexate (MTX) use

Factor	MTX (N = 9)	No MTX (N = 90)	*P*	*P*(Adjusted)
Systolic BP (mm Hg)	133.4 ± 19.9	118.2 ± 17.0	0.01	0.047
Diastolic BP (mm Hg)	79.7 ± 16.1	73.2 ± 13.5	0.16	0.33
HDL cholesterol (mg/dL)	43.2 ± 13.9	48.4 ± 14.8	0.28	0.46
LDL cholesterol (mg/dL)	123.2 ± 28.2	103.6 ± 37.8	0.06	0.23
Triglycerides (mg/dL)	161.7 ± 56.2	115.2 ± 58.3	0.02	0.05
Glucose (mg/dL)	88.1 ± 12.5	87.0 ± 28.1	0.25	0.96
Homocysteine(umol/L)	11.22 ± 3.07	9.09 ± 2.69	0.03	0.08
F_2_ Isoprostane (ng/mg Creatinine)	3.11 ± 3.30	2.80 ± 2.24	0.87	0.85

*P* = unadjusted *P* values; *P* (adjusted) = adjusted for age, sex, and SLEDAI. Data are shown as mean ± SD.

## Discussion

The major finding of this study is that current exposure to drugs used to treat SLE is not a major contributor to the cardiovascular risk factors studied.

### Corticosteroids

Systemic corticosteroids increase concentrations of triglycerides and blood pressure. Corticosteroids are thought to induce dyslipidemia through increased production of HDL, impaired catabolism of LDL, and increased lipoprotein lipase activity [3]. They also induce hypertension by increased systemic vascular resistance, extracellular volume, and cardiac contractility [3]. Corticosteroids are also known to induce glucose intolerance [13]. This study indicates that current corticosteroid use in patients with SLE is associated with higher triglyceride concentrations, but not with increased blood pressure or serum glucose concentrations. We have previously shown that cumulative exposure to corticosteroids was associated with higher triglyceride concentrations [14].

### Antimalarials

Use of antimalarials may decrease total and LDL cholesterol and triglyceride concentrations, and decreases the risk of thrombosis in patients with SLE [6,15,16]. In this study current antimalarial use was not associated with significant differences in lipid concentrations or cardiovascular risk factors. A potential explanation that should be considered is a type II error (i.e., lack of statistical power). However, based on previous data [6] reporting that antimalarials decreased LDL concentrations by approximately 26 mg/dL, a post-hoc power analysis using PS [17] showed that our sample size had approximately 93% power to detect an effect of similar magnitude. Thus, lack of statistical power is an unlikely explanation. We have previously reported that cumulative hydroxychloroquine use was not associated with lipid concentrations in this cohort of patients [14].

### NSAIDs and COX-2 Inhibitors

Traditional NSAIDs, and particularly COX-2 inhibitors, increase cardiovascular risk [4,5]. One mechanism by which NSAIDs and COX-2 inhibitors can increase cardiovascular risk is by increasing blood pressure. However, blood pressure did not differ among patients receiving NSAIDs or COX-2 inhibitors and those who did not. Triglyceride concentrations were lower in patients taking COX-2 inhibitors, but when additional statistical adjustment for corticosteroid use was performed, the association was no longer significant (*P* = 0.08). There is some experimental [18,19] and clinical evidence [20] that COX-2 inhibitors may be associated with a decrease in total cholesterol, LDL and triglyceride concentrations. Current use of COX-2 inhibitors was associated with lower F_2_-isoprostane excretion, indicative of lower oxidative stress. Other studies have suggested that COX-2 selective drugs can decrease oxidative stress [21,22].

### Methotrexate

Methotrexate is widely used to treat rheumatoid arthritis and there are several reports indicating that it increases serum homocysteine concentrations due to folate antagonism [23,24]. Concordant with those findings, this study showed an association between current use of methotrexate and increased serum homocysteine concentrations that was of marginal significance after adjustment for sex, age and SLEDAI. Since only 9 patients were receiving methotrexate, a larger sample would be required to further examine this observation. The modestly statistically significant differences in systolic blood pressure and triglycerides may be the result of the non-random design since these do not appear to be a feature of methotrexate therapy in large studies of patients with rheumatoid arthritis [25,26].

## Limitations

This study has several limitations. First, it was cross-sectional, and the pattern of drug use may have been affected by indication bias. Thus, patients with hypertension, for example, would be less likely to continue to receive drugs such as NSAIDs. However, despite this limitation, the study provides valuable information about the relationship between current use of drugs and cardiovascular risk factors. For example, in this same cohort of patients, blood pressure was significantly higher than that of controls [2], however the findings of the current study suggest that this is not explained by current exposure to NSAIDs, COX-2 selective drugs, or corticosteroids. The second limitation is that drugs would be prescribed differentially according to SLE activity. We performed statistical adjustment for current disease activity using the SLEDAI score and therefore such bias may be reduced. Third, since the study was exploratory, we have performed multiple statistical comparisons without statistical adjustment and should be considered when interpreting the results. Nevertheless, since randomized controlled trials to examine the effects of the drugs of interest on cardiovascular risk factors in SLE are not feasible, the study provides useful information, despite its limitations.
